# Prevalence of infection by the microsporidian *Nosema* spp. in native bumblebees (*Bombus* spp.) in northern Thailand

**DOI:** 10.1371/journal.pone.0213171

**Published:** 2019-03-07

**Authors:** Chainarong Sinpoo, Terd Disayathanoowat, Paul H. Williams, Panuwan Chantawannakul

**Affiliations:** 1 Bee Protection Laboratory, Department of Biology, Faculty of Science, Chiang Mai University, Chiang Mai, Thailand; 2 Graduate School, Chiang Mai University, Chiang Mai, Thailand; 3 Department of Life Sciences, Natural History Museum, London, United Kingdom; 4 Environmental Science Research Center (ESRC), Faculty of Science, Chiang Mai University, Thailand; Sichuan University, CHINA

## Abstract

Bumblebees (tribe Bombini, genus *Bombus* Latreille) play a pivotal role as pollinators in mountain regions for both native plants and for agricultural systems. In our survey of northern Thailand, four species of bumblebees (*Bombus (Megabombus) montivagus* Smith, *B*. *(Alpigenobombus) breviceps* Smith, *B*. *(Orientalibombus) haemorrhoidalis* Smith and *B*. *(Melanobombus) eximius* Smith), were present in 11 localities in 4 provinces (Chiang Mai, Mae Hong Son, Chiang Rai and Nan). We collected and screened 280 foraging worker bumblebees for microsporidia (*Nosema* spp.) and trypanosomes (*Crithidia* spp.). Our study is the first to demonstrate the parasite infection in bumblebees in northern Thailand. We found *N*. *ceranae* in *B*. *montivagus* (5.35%), *B*. *haemorrhoidalis* (4.76%), and *B*. *breviceps* (14.28%) and *N*. *bombi* in *B*. *montivagus* (14.28%), *B*. *haemorrhoidalis* (11.64%), *and B*. *breviceps* (28.257%).

## Introduction

Bumblebees (tribe Bombini, genus *Bombus* Latreille) play a vitally important role as native pollinators in temperate agricultural ecosystems [1–5]. They are especially important in mountain ecosystems [6] and may be better pollinators than honey bees for many plant species in these areas [7]. Because of this, some species of bumblebees have been employed commercially, especially in greenhouses [3]. From the 1980s onwards, they have been used commercially in greenhouses to pollinate tomatoes, eggplants, and strawberries and also for fruit trees [3, 8]. Several species have been used commercially around the world, including *Bombus terrestris*, *B*. *lucorum*, *B*. *occidentalis*, *B*. *ignitus* and *B*. *impatiens* [3, 9, 10]. Some bumblebees species (*B*. *terrestris*, *B*. *ruderatus*, *B*. *hortorum*, and *B*. *subterraneus*) had been released in New Zealand for targeted pollination in the 19th century [11]. Among species used commercially, the most frequent are *B*. *terrestris* in Europe and *B*. *impatiens* in North America [3]. The identification of bumblebee species has been difficult because the colour patterns can be highly variable within species and convergent among species [12].

In recent years, molecular approaches have been applied for bumblebee identification using particularly a mitochondrial gene (cytochrome oxidase I (COI)) [7]. COI barcodes provide an easily obtained, dependable and cost-effective solution, especially for morphologically cryptic species [13]. Consequently, the COI gene has been used to re-evaluate species, to estimate phylogenetic relationships and to clarify species complexes in Asian bumblebees [14–18].

Similar to *Apis* bees, bumblebee populations are affected by a number of pathogens and parasites [19]. *Crithidia bombi* (Trypanosomatidae) and *Nosema bombi* are the most common. They are transmitted both horizontally between and vertically within colonies of their hosts [20]. *Nosema bombi* (Microsporidia: Nosematidae) is an obligate intracellular microsporidian parasite infecting a wide range of bumblebee species [20–24]. It is the most widespread bumblebee pathogen worldwide. Thorp (2005) suggested that *N*. *bombi*, known to infect European *Bombus* species [25], may have invaded North American species [25]. Imhoof et al. (1999) showed that prevalence of *N*. *bombi* was significantly higher in two declining species, *B*. *pensylvanicus* and *B*. *occidentalis*, than in other species [26]. In addition, *Nosema cerana* and *C*. *bombi* are associated with declining populations of bumble bees in China [27].

In this paper, we aim to study the diversity of native bumblebees in northern Thailand and to report the prevalence of microsporidians and trypanosomes parasitizing bumblebee populations in Thailand.

## Materials and methods

The sample locations for which specific permission was not required and bumblebee did not involve endangered or protected species.

### Collection and sample preparation

**Fig 1:**
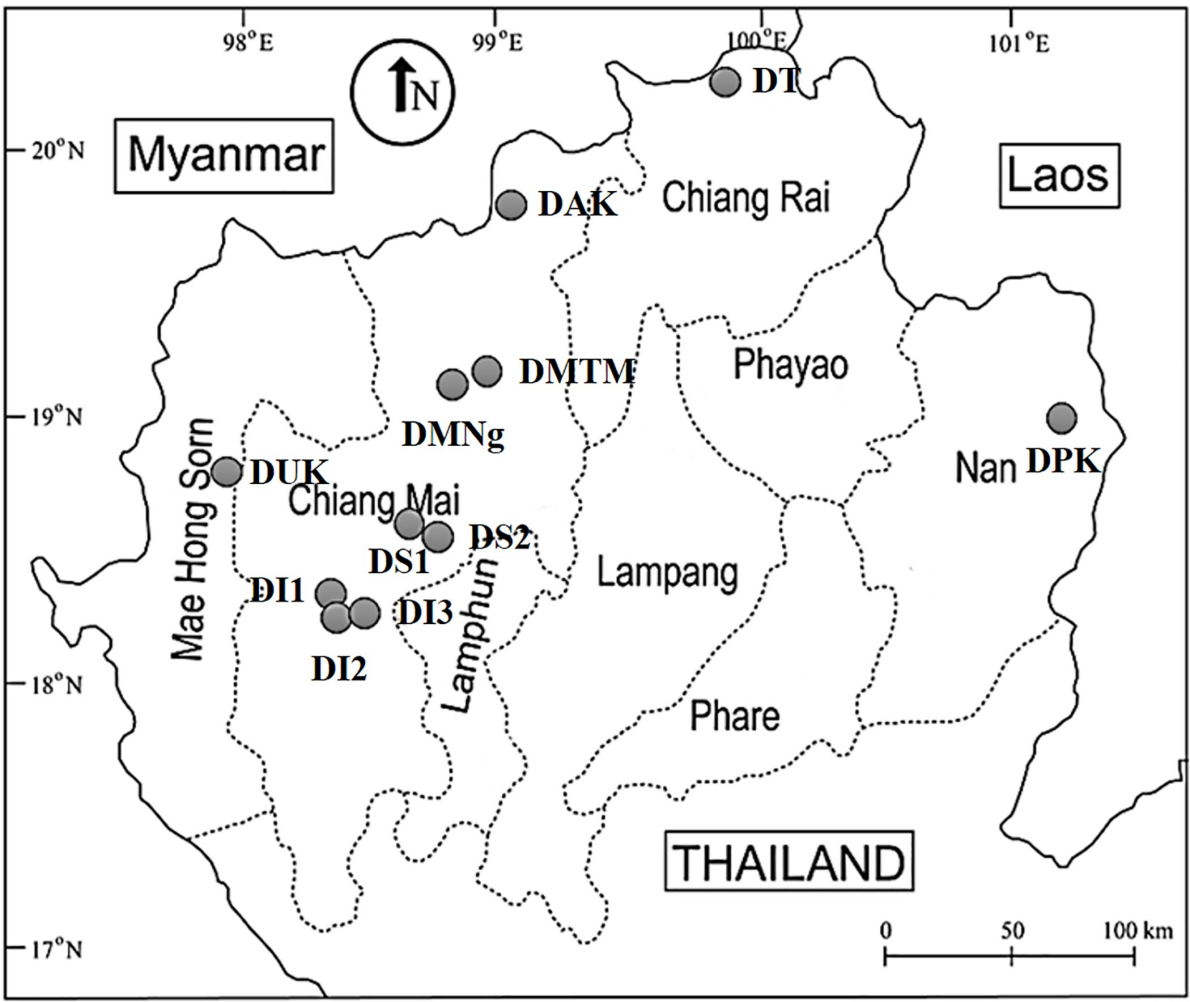
Map of the collection sites (grey dots) of native bumblebees in northern Thailand. Code name are abbreviated as following: DS = Doi Suthep, DI = Doi Inthanon, DMTM = Doi Mae Thaman, DAK = Doi Ang Khang, DMNg = Doi Mon Ngao, DUK = Doi Mae U Kho, DT = Doi Thong, DPK = Doi Phu Kha.

Foraging bumblebees were collected with sweep nets and as random samples from seven sites in four provinces in northern Thailand (Chiang Mai, Mae Hong Son, Chiang Rai and Nan province) in 2015 & 2016 (Table 1). After capture, they were transferred directly into RNA *later* Solution and stored at -20°C prior to DNA extraction. The following information was recorded for each specimen: GPS coordinates, elevation, collection-site name, and date. The samples were later analyzed in the laboratory. The exact locations are listed in Table 1 and shown in Fig 1. Bumblebee taxa were identified using an updated version of the morphological characters of Williams (2010) [28].

**Table 1:** Prevalence of four parasites recovered from *Bombus* species in northern Thailand.

Province population	Code Name	Elevation	Latitude N	Longitude E	N Bees collected	Prevalence of parasites (%)			
						*N*. *apis*	*N*. *ceranae*	*N*. *bombi*	*C*. *bombi*
CHIANG MAI									
Doi Suthep 1	DS1	1,378	18°48′55"	98°55′13"	60	0.00	3.33	10.00	0
Doi Suthep 2	DS2	1,378	18°48′55"	98°55′13"	20	0	5.00	15.00	0
Doi Inthanon 1	DI1	2,118	18°33′11"	98°28′55"	25	0	0	12.00	0
Doi Inthanon 2	DI2	1,297	18°32′41"	98°30′58"	40	0	7.50	20.00	0
Doi Inthanon 3	DI3	1,070	18°32′38"	98°32′53"	40	0	12.50	15.00	0
Doi Mae Tha Man	DMTM	1,610	19°31′35"	98°83′26"	5	0	0	20.00	0
Doi Ang Khang	DAK	1,410	19°54′8"	99°2′24"	25	0	4.00	8.00	0
Doi Mon Ngao	DMNg	930	19°10′60"	99°48′35"	20	0	0	15.00	0
MAE HONG SON									
Doi Mae U Kho	DUK	1,509	18°53′41"	98°05′21"	20	0	10.00	20.00	0
CHIANG RAI									
Doi Thong	DT	960	20°17′18"	99°48′35"	20	0	5.00	20.00	0
Nan									
Doi Phu Kha	DPK	1,980	19°12′20″	101°40′50"	5	0	20.00	0	0
				Total	280	0	5.71	13.57	0

### DNA extraction, mitochondrial cytochrome oxidase 1 (COI) gene sequence amplification

DNA extraction was achieved using a single crushed mid leg from each of the bumblebees. For most specimens, legs were ground in a 0.5-mL oxygen tube in liquid nitrogen using a stainless steel pestle, a Proteinase K Digestion kit was used, and the DNA was extracted following a standard phenol-chloroform protocol [29]. DNA extracts were kept at -20°C until needed as a DNA template for the PCR (polymerase chain reaction). The PCR products of the mitochondrial COI (~685 base pairs) sequence were conducted using the universal primers LCO1490 and HC02198 [30]. The PCR amplification was performed in a total volume of 25 μL containing 2 μL of DNA extract, 12.5 pM of each primer, 0.2 mM of each dNTP, 0.2 mM MgCl_2_, 1X reaction buffer and 2.5 units of *Taq* DNA polymerase (Invitrogen) under the following thermal conditions: 94°C for 1 min, 5 cycles of 94°C for 1 min, 50°C for 1.5 min, 72°C for 1 min; 35 cycles of 94°C for 1 min, 50°C for 1.5 min, 72°C for 1 min and final step 72°C for 5 min. Amplicons were checked on 1% agarose gels stained with ethidium bromide under UV light. PCR products were purified using PureLink Quick PCR Purification Kit (Invitrogen, Lithuania, USA) following the manufacturer's protocol. The purified PCR products were sequenced. Sequencing reactions were performed, and the sequences were automatically determined in a genetic analyzer (1^st^ Base, Selangor, Malaysia) using PCR primers mentioned above.

### DNA Isolation and PCR Detection for pathogen/parasite

The abdomens of 280 individual bumblebees were removed with scissors and individually homogenized in 100 μL of Krebs-Ringer solution with a sterile Eppendorf tube. Total genomic DNA was extracted from 50 μL of the homogenate of each abdomen using a DNA purification kit (PureLink Genomic DNA Mini Kit (Invitrogen)). DNA samples were stored at -20°C prior to molecular screening for parasites. Primers used for detection of *N*. *ceranae*, *N*. *apis*, *N*. *bombi* and *C*. *bombi* are listed in Table 2. The PCR amplification was performed in a total volume of 25 μL containing 2 μL DNA extract, 12.5 pM of each primer, 0.2 mM of each dNTP, 0.2 mM MgCl_2_, 1X reaction buffer and 2.5 unit of Taq DNA polymerase (Invitrogen). Amplification used thermal cycling profiles: initial DNA denaturation step of 4 min at 94°C followed by 40 cycles of 30s at 94°C, 30s at 56°C, and 1 min at 72°C, and terminated with a final extension step of 72°C for 10 min. For each run of the PCR reaction, negative (water) and positive (previously identified positive sample) controls were run along with DNA extracts of the samples. PCR products were electrophoresed on 1.2% agarose gels with ethidium bromide and visualized under UV light. Some of the PCR-amplified bands were purified with PureLink Quick PCR Purification Kit (Invitrogen, Lithuania, USA) following the manufacturer's protocol. After the sequencing reactions the sequences were determined automatically in a genetic analyzer (1^st^ Base, Selangor, Malaysia) using the PCR primers mentioned above. The DNA sequences were used for estimating phylogenetic trees.

**Table 2:** Primers used for pathogen/parasite and mtDNA detection.

Primer	Sequence 5′-3′	Amplification target	Size (bp)	Reference
**RPS5-F**	AATTATTTGGTCGCTGGAATTG	Ribosomal protein S5 (reference gene)		Evans (2006)[31]
**RPS5-R**	TAACGTCCAGCAGAATGTGGTA			
**LCO1490**	GGTCAACAAATCATAAAGATATTGG	mtDNA	685	Folmer et al. (1994)[30]
**HCO2198**	TAAACTTCAGGGTGACCAAAAAATCA			
**Crith-F**	GGAAACCACGGAATCACATAGACC	*Crithidia* (Trypanosome)	500	Li et al. (2012)[32]
**Crith-R**	AGGAAGCCAAGTCATCCATCGC			
**Napis-SSU-Jf1**	CCATGCATGTCTTTGACGTACTATG	*N*.*apis* (Microsporidium)	325	Klee et al. (2007)[33]
**Napis-SSU-Jr1**	GCTCACATACGTTTAAAATG			
**NOS-FOR**	TGCCGACGATGTGATATGAG	*N*.*ceranae* (Microsporidium)	252	Higes et al. (2006)[34]
**NOS-REV**	CACAGCATCCATTGAAAACG			
**Nbombi-SSU-Jf1**	CCATGCATGTTTTTGAAGATTATTAT	*N*. *bombi* (Microsporidium)	323	Klee et al. (2007)[33]
**Nbombi-SSU-Jr1**	CATATATTTTTAAAATATGAAACAATAA			

### Data analysis

Sequences were checked manually and aligned using the BioEdit (version v7.2.6; http://www.mbio.ncsu.edu/BioEdit/BioEdit.html, accessed 2017), and the primers removed from both ends (Table 2). The sequences were aligned using ClustalW and the alignments were refined by visual inspection. Sequences were used to query GenBank via the BLAST program (https://blast.ncbi.nlm.nih.gov/Blast.cgi). All covering DNA cytochrome oxidase I (COI) region and *Nosema* parasites sequences obtained in this study can be accessed as NCBI GenBank entries (http://www.ncbi.nlm.nih.gov; bumblebee species accession number MF582589—MF582628; *Nosema* parasites accession number MF776532-MF776567).

For phylogenetic analysis, multiple alignments of sequences determined in this study and reference sequences obtained from databases were taken together in the calculations of levels of sequence similarity using ClustalX2 program [35], with arithmetic averages tree-making algorithms taken from the MEGA package version 7 [36]. The topologies of the maximum likelihood phylogenetic trees were evaluated based on bootstrap analyses of 1,000 replicates.

## Results

### Geographical distribution

Samples were collected from Chiang Mai, Mae Hong Son, Chiang Rai and Nan province, at an elevation range of 700‒2,200 m. (sample site; Fig 1, Table 1 and Table 3).

**Table 3:** A list of *Bombus* subgenera with information on distribution and species number.

Subgenus	Distribution	Species	No. sampled
*Alpigenobombus*	DS1. DS2 DI1, DI2, DI3	*B*. *breviceps*	28
*Megabombus*	DS1, DI2, DI3, DAK, DUK	*B*. *montivagus*	56
*Melanobombus*	DI1	*B*. *eximius*	7
*Orientalibombus*	DS1,DS2, DI2, DT, DAK, DMNg, DPK	*B*. *haemorrhoidalis*	189

**Fig 2:**
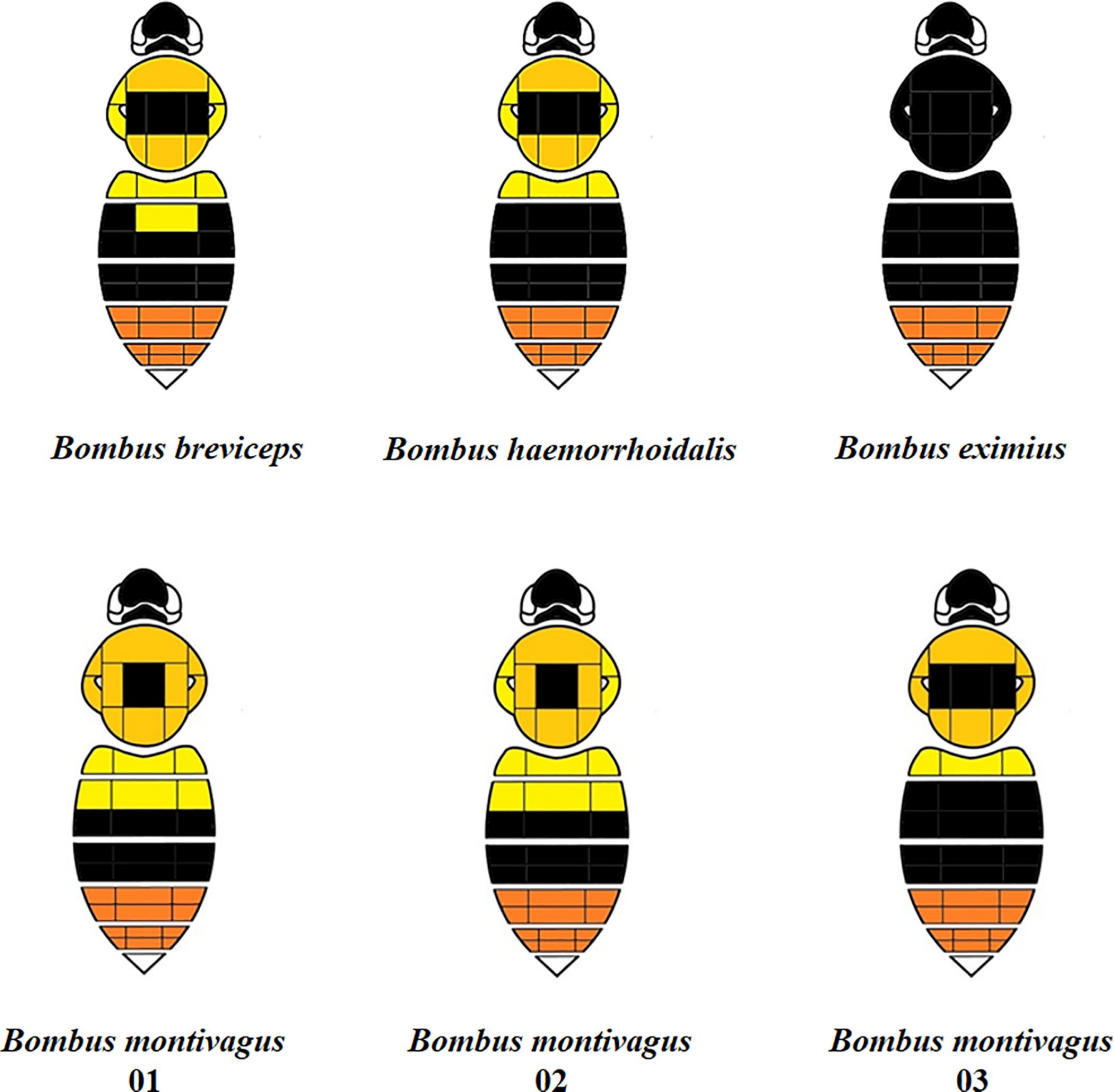
Species identification guide with simplified colour patterns of female workers. The dorsum of the body is artificially divided into an arbitrary set of regions.

Our study of bumblebees in northern Thailand included 280 female bumblebees. Many of the bumblebees’ colour patterns were similar among species within northern Thailand. The dominant colour of the 6^th^ abdominal segment was red in all of the specimens. Of *B*. *montivagus*, three distinct colour patterns were collected (Fig 2). In this study, similar colour patterns to those of *B*. *montivagus* were observed in co-occurring species, *B*. *haemorrhoidalis* and *B*. *breviceps*. The colour pattern of the thoracic pubescence of the workers was primarily orange. In *B*. *breviceps*, *B*. *haemorrhoidalis*, *and B*. *montivagus*, the described orange colour pattern runs anterior to posterior on the notum of the thorax. However, some species have extensive black hair on the thorax, ranging from a small patch in the center of the thorax to a transverse band between the tegulae (above the wing bases), or (in the case of *B*. *eximius*) the entire thorax. The sides of the thorax are orange or yellow in all species except *B*. *eximius*.

### COI-sequence-based analyses

DNA was extracted and the COI gene sequence was amplified successfully from 40 individual bumblebee specimens from 11 localities. All of the sequences were 658 base pairs long after removing the primer from both ends. We found a strong A+T bias in the COI gene barcoding from mtDNA. All new sequences have been deposited in GenBank and are accessible via the sequence numbers MF582589‒MF582628 (Table 4).

**Table 4:** Material used in the phylogenetic analysis with the sample localities, collector, COI sequence length, depository and GenBank accession number.

Species	Sample name	Sample locality	Collector	Latitude	Longitude	Sequence length (bp)	GenBank acc. no.
*Montivagus*	DS1-B01	TH, Doi Su Thep CMP	C. Sinpoo	18°48′55"	98°55′13"	658	MF582589
*haemorrhoidalis*	DS1-B16	TH, Doi Su Thep CMP	C. Sinpoo	18°48′55"	98°55′13"	658	MF582590
*haemorrhoidalis*	DS1-B21	TH, Doi Su Thep CMP	C. Sinpoo	18°48′55"	98°55′13"	658	MF582591
*haemorrhoidalis*	DS1-B41	TH, Doi Su Thep CMP	C. Sinpoo	18°48′55"	98°55′13"	658	MF582592
*montivagus*	DI2-B06	TH, Doi Inthanon CMP	C. Sinpoo	18°32′41"	98°30′58"	658	MF582593
*haemorrhoidalis*	DI2-B16	TH, Doi Inthanon CMP	C. Sinpoo	18°32′41"	98°30′58"	658	MF582594
*haemorrhoidalis*	DI2-B31	TH, Doi Inthanon CMP	C. Sinpoo	18°32′41"	98°30′58"	658	MF582595
*montivagus*	DI3-B11	TH, Doi Inthanon CMP	C. Sinpoo	18°32′38"	98°32′53"	658	MF582596
*montivagus*	DI3-B21	TH, Doi Inthanon CMP	C. Sinpoo	18°32′38"	98°32′53"	658	MF582597
*breviceps*	DI3-B27	TH, Doi Inthanon CMP	C. Sinpoo	18°32′38"	98°32′53"	658	MF582598
*haemorrhoidalis*	DMNg-B01	TH, Doi Mon Ngao CMP	C. Sinpoo	19°10′60"	99°48′35"	658	MF582599
*haemorrhoidalis*	DMNg-B11	TH, Doi Mon Ngao CMP	C. Sinpoo	19°10′60"	99°48′35"	658	MF582600
*haemorrhoidalis*	DAK-B01	TH, Doi Ang Khang CMP	C. Sinpoo	19°54′8"	99°2′24"	658	MF582601
*haemorrhoidalis*	DAK-B14	TH, Doi Ang Khang CMP	C. Sinpoo	19°54′8"	99°2′24"	658	MF582602
*montivagus*	DAK-B05	TH, Doi Ang Khang CMP	C. Sinpoo	19°54′8"	99°2′24"	658	MF582603
*haemorrhoidalis*	DAK-B12	TH, Doi Ang Khang CMP	C. Sinpoo	19°54′8"	99°2′24"	658	MF582604
*montivagus*	DAK-B22	TH, Doi Ang Khang CMP	C. Sinpoo	19°54′8"	99°2′24"	658	MF582605
*haemorrhoidalis*	DAK-B06	TH, Doi Ang Khang CMP	C. Sinpoo	19°54′8"	99°2′24"	658	MF582606
*haemorrhoidalis*	DAK-B10	TH, Doi Ang Khang CMP	C. Sinpoo	19°54′8"	99°2′24"	658	MF582607
*montivagus*	DUK-B01	TH, Doi Mae U Kho MHP	C. Sinpoo	18°53′41"	98°05′21"	658	MF582608
*montivagus*	DUK-B08	TH, Doi Mae U Kho MHP	C. Sinpoo	18°53′41"	98°05′21"	658	MF582609
*haemorrhoidalis*	DT-B01	TH, Doi Thong CRP	C. Sinpoo	20°17′18"	99°48′35"	658	MF582610
*haemorrhoidalis*	DT-B04	TH, Doi Thong CRP	C. Sinpoo	20°17′18"	99°48′35"	658	MF582611
*breviceps*	DI2-B20	TH, Doi Inthanon CMP	C. Sinpoo	18°32′41"	98°30′58"	658	MF582612
*breviceps*	DI3-B30	TH, Doi Inthanon CMP	C. Sinpoo	18°32′38"	98°32′53"	658	MF582613
*haemorrhoidalis*	DS2-B01	TH, Doi Su Thep CMP	C. Sinpoo	18°48′55"	98°55′13"	658	MF582614
*haemorrhoidalis*	DS2-B02	TH, Doi Su Thep CMP	C. Sinpoo	18°48′55"	98°55′13"	658	MF582615
*haemorrhoidalis*	DS2-B03	TH, Doi Su Thep CMP	C. Sinpoo	18°48′55"	98°55′13"	658	MF582616
*breviceps*	DS2-B04	TH, Doi Su Thep CMP	C. Sinpoo	18°48′55"	98°55′13"	658	MF582617
*breviceps*	DI3-B01	TH, Doi Inthanon CMP	C. Sinpoo	18°32′38"	98°32′53"	658	MF582618
*Breviceps*	DI3-B03	TH, Doi Inthanon CMP	C. Sinpoo	18°32′38"	98°32′53"	658	MF582619
*montivagus*	DI2-B01	TH, Doi Inthanon CMP	C. Sinpoo	18°32′41"	98°30′58"	658	MF582620
*haemorrhoidalis*	DI2-B03	TH, Doi Inthanon CMP	C. Sinpoo	18°32′41"	98°30′58"	658	MF582621
*Breviceps*	DI2-B04	TH, Doi Inthanon CMP	C. Sinpoo	18°32′41"	98°30′58"	658	MF582622
*haemorrhoidalis*	DI2-B05	TH, Doi Inthanon CMP	C. Sinpoo	18°32′41"	98°30′58"	658	MF582623
*haemorrhoidalis*	DPK-B01	TH, Doi Phu Kha NP	C. Sinpoo	19°12′20″	101°40′50"	658	MF582624
*haemorrhoidalis*	DPK-B02	TH, Doi Phu Kha NP	C. Sinpoo	19°12′20″	101°40′50"	658	MF582625
*eximius*	DI1-B02	TH, Doi Inthanon CMP	C. Sinpoo	18°33′11"	98°28′55"	658	MF582626
*eximius*	DI1-B03	TH, Doi Inthanon CMP	C. Sinpoo	18°33′11"	98°28′55"	658	MF582627
*Breviceps*	DI1-B04	TH, Doi Inthanon CMP	C. Sinpoo	18°33′11"	98°28′55"	658	MF582628

**Fig 3:**
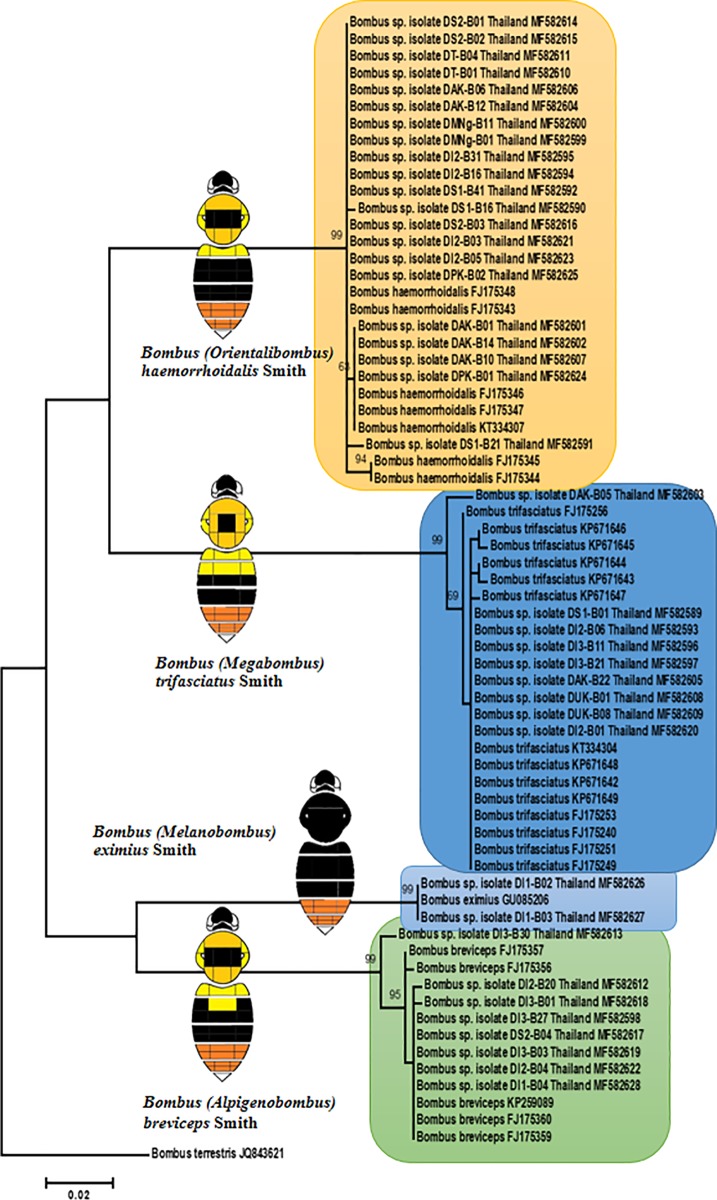
Estimate of phylogenetic relationship of cytochrome oxidase subunit I (COI) from bumblebees (*Bombus* sp.) collected in northern Thailand using maximum likelihood. The sequences of *B*. *terrestris*–JQ843621 was used as an out group. Numbers at each node represent bootstrap values as percentages and only bootstrap values greater than 70% are shown.

The phylogenetic analysis by maximum likelihood method (Fig 3) with COI barcode data showed strong support for all of the following four conventional *Bombus* subgenera: *B*. *(Megabombus) montivagus* Smith (formerly regarded as part of *B*. *trifasciatus s*. *l*.), *B*. *(Alpigenobombus) breviceps* Smith, *B*. *(Orientalibombus) haemorrhoidalis* Smith *and B*. *(Melanobombus) eximius* Smith (Fig 3).

### Microsporidian and trypanosome parasite frequencies in bumblebees

A total of 280 individual bumblebees representing four species (*B*. *montivagus*, *B*. *haemorrhoidalis*, *B*. *breviceps*, and *B*. *eximius*) were examined from samples from northern Thailand (Chiang Mai, Mae Hong Son, Chiang Rai and Nan province, sampling sites shown in Table 1). We collected and screened for the most common pathogens of foraging worker bumblebees, *Nosema* spp. and *Crithidia* spp..

The results showed that 16 out of 280 individual bumblebees (5.71%) were infected with *N*. *ceranae*. This parasite was found in specimens of *B*. *montivagus* (5.35%), *B*. *breviceps* (14.28%), *and B*. *haemorrhoidalis* (4.76%). *Nosema bombi* was found in 38 individuals (13.57%) from the three species of *Bombus* as shown in Table 5. Infection rates of *N*. *ceranae* and *N*. *bombi* were higher in *B*. *breviceps* than in other bumblebee species. *Nosema bombi* was also more prevalent than *N*. *ceranae* in the three species of bumblebees. When considering the geographical areas, the highest prevalence values of *N*. *ceranae* (20% and 12.5% respectively) were found at the locations Doi Phu Kha (Nan) and Doi Inthanon 3 (Chiang Mai). Prevalence of *N*. *bombi* of 20% was found at Doi Inthanon 2, Doi Mae Tha Man (Chiang Mai) and Doi Mae U Kho (Mae Hong Son).

**Table 5:** Overall occurrence of four parasites in host species (*Bombus* spp.) (Identities confirmed from barcodes).

Species	N Bees collected	*N*. *apis* ^*a*^	*N*. *ceranae*^a^	*N*. *bombi*^*a*^	*C*. *bombi*^a^
*B*. *montivagus*	56	0.00	5.35	14.28	0.00
*B*. *haemorrhoidalis*	189	0.00	4.76	11.64	0.00
*B*. *breviceps*	28	0.00	14.28	28.57	0.00
*B*. *eximius*	7	0.00	0.00	0.00	0.00
Total	280	0.00	5.71	13.57	0.00

N = Total number of individual each *Bombus* species collected.

^a^ = Prevalence (%)

**Fig 4:**
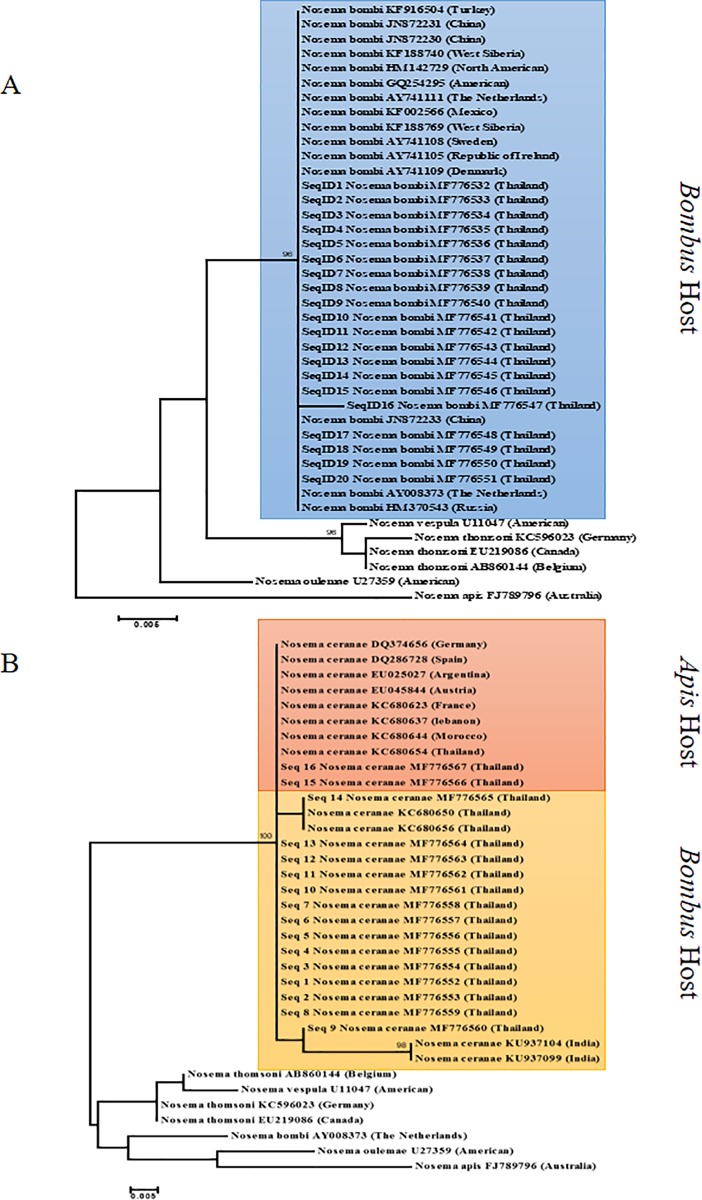
The phylogenetic tree showing the relationship of *Nosema*. Unrooted consensus of phylogenetic tree showing the relationship of *Nosema* isolate the partial sequences of 16S ribosomal RNA of *Nosema* (4-A; *N bombi*, 4-B; *N*. *ceranae*) from *Bombus* spp. collected in northern Thailand. The tree was estimated using Maximum Likelihood. Numbers at each node represent bootstrap values as percentages and only bootstrap values greater than 70% are shown.

Phylogenetic trees were estimated to assess relationships between the samples of *Nosema* as shown in Fig 4A and 4B. This included a total of 36 sequences from infected *Bombus* with a length of 269 bp for 20 sequences of *N*. *bombi* and 212 bp for 16 sequences of *N*. *ceranae*, after removing the primers from both ends. New sequences of *Nosema* have been deposited in GenBank and are accessible with the numbers MF776532–MF776567 (Table 6).

**Table 6:** Material used in the phylogenetic analysis with the sample locality, collector, sequence length, depository and GenBank accession number.

Species	Sample name	Sample locality	Collector	Sequence length (bp)	GenBank
1 *N*. *bombi*	BomDS2-B06	TH, Doi Su Thep CMP	C. Sinpoo	269	MF776532
2 *N*. *bombi*	BomDS2-B12	TH, Doi Su Thep CMP	C. Sinpoo	269	MF776533
3 *N*. *bombi*	BomDS2-B20	TH, Doi Su Thep CMP	C. Sinpoo	269	MF776534
4 *N*. *bombi*	BomDS1-B04	TH, Doi Su Thep CMP	C Sinpoo	269	MF776535
5 *N*. *bombi*	BomDS1-B10	TH, Doi Su Thep CMP	C. Sinpoo	269	MF776536
6 *N*. *bombi*	BomDS1-B37	TH, Doi Su Thep CMP	C Sinpoo	269	MF776537
7 *N*. *bombi*	BomDS1-B45	TH, Doi Su Thep CMP	C. Sinpoo	269	MF776538
8 *N*. *bombi*	BomDS1-B55	TH, Doi Su Thep CMP	C. Sinpoo	269	MF776539
9 *N*. *bombi*	BomDI1-B04	TH, Doi Inthanon CMP	C. Sinpoo	269	MF776540
10 *N*. *bombi*	BomDI1-B07	TH, Doi Inthanon CMP	C. Sinpoo	269	MF776541
11 *N*. *bombi*	BomDI1-B11	TH, Doi Inthanon CMP	C. Sinpoo	269	MF776542
12 *N*. *bombi*	BomDI2-B17	TH, Doi Inthanon CMP	C. Sinpoo	269	MF776543
13 *N*. *bombi*	BomDI2-B24	TH, Doi Inthanon CMP	C. Sinpoo	269	MF776544
14 *N*. *bombi*	BomDI3-B07	TH, Doi Inthanon CMP	C. Sinpoo	269	MF776545
15 *N*. *bombi*	BomDMNg-B05	TH, Doi Mon Ngao CMP	C. Sinpoo	269	MF776546
16 *N*. *bombi*	BomDMNg-B11	TH, Doi Mon Ngao CMP	C. Sinpoo	269	MF776547
17 *N*. *bombi*	BomDMNg-B15	TH, Doi Mon Ngao CMP	C. Sinpoo	269	MF776548
18 *N*. *bombi*	BomDMTM-B03	TH, Doi Mae Tha Man CMP	C. Sinpoo	269	MF776549
19 *N*. *bombi*	BomDAK-B10	TH, Doi Ang Khang CMP	C. Sinpoo	269	MF776550
20 N. *bombi*	BomDAK-B12	TH, Doi Ang Khang CMP	C. Sinpoo	269	MF776551
1 *N*. *ceranae*	BomDS2-B16	TH, Doi Su Thep CMP	C. Sinpoo	212	MF776552
2 N. *ceranae*	BomDS1-B10	TH, Doi Su Thep CMP	C. Sinpoo	212	MF776553
3 N. *ceranae*	BomDS1-B37	TH, Doi Su Thep CMP	C. Sinpoo	212	MF776554
4 N. *ceranae*	BomDI2-B02	TH, Doi Inthanon CMP	C. Sinpoo	212	MF776555
5 N. *ceranae*	BomDI3-B02	TH, Doi Inthanon CMP	C. Sinpoo	212	MF776556
6 N. *ceranae*	BomDAK-B10	TH, Doi Ang Khang CMP	C. Sinpoo	212	MF776557
7 N. *ceranae*	BomDUK-B10	TH, Doi Mae U Kho CMP	C. Sinpoo	212	MF776558
8 *N*. *ceranae*	BomDT-B16	TH, Doi Thong CRP	C. Sinpoo	212	MF776559
9 *N*. *ceranae*	BomDT-B16	TH, Doi Thong CRP	C. Sinpoo	212	MF776560
10 *N*. *ceranae*	BomDI2-B04	TH, Doi Inthanon CMP	C. Sinpoo	212	MF776561
11 *N*. *ceranae*	BomDI2-B38	TH, Doi Inthanon CMP	C. Sinpoo	212	MF776562
12 *N*. *ceranae*	BomDI3-B05	TH, Doi Inthanon CMP	C. Sinpoo	212	MF776563
13 *N*. *ceranae*	BomDI3-B23	TH, Doi Inthanon CMP	C. Sinpoo	212	MF776564
14 *N*. *ceranae*	BomDI3-B27	TH, Doi Inthanon CMP	C. Sinpoo	212	MF776565
15 *N*. *ceranae*	BomDI3-B39	TH, Doi Inthanon CMP	C. Sinpoo	212	MF776566
16 *N*. *ceranae*	BomDUK-B19	TH, Doi Inthanon MHS	C. Sinpoo	212	MF776567

## Discussion

In this study we aimed to identify native bumblebees from multiple sites in northern Thailand (Chiang Mai, Mae Hong Son, Chiang Rai and Nan province). Three bumblebee species (*B*. *montivagus* Smith, *B*. *haemorrhoidalis* Smith, and *B*. *breviceps* Smith) show similar colour patterns. These colour patterns are similar to others in Southeast Asia and may have evolved though mutually protective Mullerian mimicry [37]. We have identified similar colour patterns for bumblebee workers (Fig 2) (three of them for *B montivagus* in northern Thailand). Hines and Williams (2012) examined colour-pattern evolution in bumblebees in this Southeast Asian mimicry group, which includes *B*. (*Megabombus*) *montivagus* Smith, *B*. (*Alpigenobombus*) *breviceps* Smith, and *B*. (*Orientalibombus*) *haemorrhoidalis* Smith [37]. Moreover, they reported that because these bumblebees also have high variability of colour patterns within species it is sometimes difficult to make reliable species identifications. Considerable colour variation within bumblebee species has been known for more than a century [38]. Our work reaffirms that only some morphological data can be used to accurately distinguish species.

When possible, additional molecular data should therefore be used to confirm species identification [15, 37, 39, 40]. According to our results, the bumblebee species are supported by groups identified from the (COI) gene. This confirms the value of evidence from barcodes for examining the more closely related bumblebee species despite the variation within species [15, 40, 41].

This study is the first survey of the prevalence of major bumblebee pathogens in native bumblebees in northern Thailand, showing the detection and infection rates of *N*. *cerana* and *N*. *bombi* among 280 female bumblebee specimens. In this sample, *N*. *bombi* was present in three species of *Bombus* (i.e. *B*. *montivagus*, *B*. *haemorrhoidalis*, and *B*. *breviceps*). The complete gene encoding ssrRNA sequences of *Nosema* isolates were identical to those reported previously from the bumblebee species *B*. *terrestris*, *B*. *hortorum*, and *B*. *lucorum* [21]. Cameron et al. (2011) and Kissinger et al. (2011) could only analyze *N*. *bombi* in samples of various *Bombus* spp. from the southern states of the USA, which were genetically similar to the European isolates screened by these authors [5, 42]. In our results, the gene sequences showed small variations. In the past it was believed that among all *Nosema* taxa identified to date, only *N*. *bombi* was an established parasite of *Bombus* spp. [21] in which it may be present at varying levels [19, 43]. Thorp (2005) and Tay et al. (2005) suggested that *N*. *bombi* was the only microsporidian known to infect European *Bombus* species [20, 25].

Our study found that *N*. *ceranae* was also present in three *Bombus* spp. (*B*. *montivagus*, *B*. *haemorrhoidalis*, and *B*. *breviceps)*. Normally, *N*. *ceranae* infects honey bees (originally isolated from *A*. *cerana* [44] now infecting *A*. *mellifera* as well [33, 45]), but Plischuk et al (2009) found *N*. *ceranae* in bumblebees in South America [46]. Our work also is similar to the findings of researchers who have reported the presence of *N*. *ceranae* in native bumblebees of Argentina (*B*. *atratus*, *B*. *bellicosus*, and *B*. *morio*) [46]. Mean prevalence values of *N*. *ceranae* found in *B*. *breviceps* (14.28%) are lower than those reported in *B*. *atratus* (72%) and *B*. *bellicosus* (63%) from Argentina [47] as well as from these same species in other countries [32, 48]. On the other hand, the lower infection intensity found in native bumblebees of northern Thailand may prevent infection from increasing further as natural reservoirs with high prevalence of the pathogen have not yet been found.

We collected and screened the most common pathogens for total of 280 native foraging worker bumblebees. The trypanosome *C*. *bombi* was not observed in this study. Kissinger et al. (2011) also reported few *C*. *bombi* in his extensive survey [42]. Similarly, prevalence of *Crithidia* was less than 10% of all *Bombus* species examined in United States [49].

Previous studies have proposed that *N*. *ceranae* is closer phylogenetically to *N*. *bombi* than to *N*. *apis* [21, 50, 51], although there is a report to the contrary [52]. Shafer et al. (2009) suggest that *N*. *apis* is a basal member of the clade and, therefore, *N*. *bombi* is closer to *N*. *ceranae* [53]. In our study, *N*. *ceranae* strains present in three species of *Bombus* (*B*. *montivagus*, *B*. *haemorrhoidalis*, and *B*. *breviceps*) from northern Thailand were closely related to the *N*. *ceranae* strains reported from *A*. *mellifera*. This reaffirms that *N*. *ceranae* has a broad host range and may cross between host genera. *Nosema ceranae* was first discovered in *A*. *cerana*, however although it is now spreading to *A*. *mellifera*. This pathogen has potential as an emerging threat to bumblebees among the indigenous pollinators [54].
